# Are we adequately training our healthcare providers facing the pandemic of obesity?

**DOI:** 10.20945/2359-4292-2024-0272

**Published:** 2024-07-18

**Authors:** Eric Ravussin

**Affiliations:** 1 Pennington Biomedica l Research Foundation Baton Rouge Louisiana USA Pennington Biomedica l Research Foundation,Baton Rouge, Louisiana, USA

During the second part of the 20^th^ century, public health systems in collaboration with pharmaceutical companies have made enormous progress in curbing the mortality from infectious disease in both westernized and developing countries. However, the tremendous economic development after the second world war triggered another health epidemic linked to changes in lifestyle characterized by increasing availability of food and decreasing needs in occupational-related physical activity. As countries grow economically and healthcare improves, people live longer, but chronic conditions like obesity, insulin resistance, heart disease, diabetes and many forms of cancers become more common. This complex transition affects global health dynamics with infectious diseases being replaced by chronic diseases of aging now draining public health resources to treat them. In 2022, one in 8 people in the world were living with obesity (1). The prevalence of adult obesity has more than doubled since 1990 and that in adolescents has quadrupled. Even more alarming, after the impressive progress in life expectancy during the 20^th^ century, it is now decreasing due to obesity and its associated diseases (2). Alarmingly, in Asia and Africa, infectious diseases are being replaced by chronic diseases. This shift is of course tied to aging populations but importantly to economic developments across these regions. Many countries are presently facing a dual burden of undernutrition and overweight/obesity, both bankrupting public health resources (3).

Several epidemiological and clinical studies have clearly identified three important lifestyles factors underlying health and disease: nutrition, physical activity levels, and sleep hygiene, all of which can accelerate or slow down the rate of biological aging. For centuries, we have known that nutrition can adversely impact health (many nutritional insufficiencies) but also improve it (balance nutrient rich diets). In the eyes of Hippocrates, an ancient Greek physician who lived around 400 BC, nutrition was recognized as an important factor in good health. His famous quote “Let food be thy medicine and medicine be thy food” tells it all. Over the past two decades or so, it has been recognized that food is medicine but very little has been done to better train and educate health care providers to advise patients and populations on how to better eat in our obesogenic environment. This would represent a first step towards the maintenance of healthier weights in individuals and populations and therefore promote better health.

In this issue of the Journal, Vasques and cols. present a compelling argument for the instalment of “Culinary Medicine” in medical schools around the world. In an article entitled “*Cooking for Health: a comprehensive narrative review of culinary medicine as an educational tool in medical training in Brazil and globally*”, the authors describe (4):

1. the role of nutrition in health and disease.
2. the foundation of Culinary Medicine including “physician’s self-care, culinary skills, the principles of nutrition, and communication to patients”.
3. the role of healthcare professionals in providing nutritional guidance to their patients.
4. the state of teaching Culinary Medicine in Brazil and other countries.

The authors clearly state the lack of training of healthcare professionals when it comes to the role of nutrition in health and diseases. With the rising prevalence of obesity around the world and its deleterious impact on chronic disease of aging reducing life expectancy, it is now imperative to expose physicians and healthcare professionals to the importance of nutrition in delaying the incidence of health problems related to poor lifestyle. Even if new elective courses in Culinary Medicine are emerging in medical schools around the world, it is still at a very preliminary level. There is now a need to add Culinary Medicine to the curriculum of medical schools. It is imperative to improve the dialogue between physicians and healthcare professionals when it comes to prescribing better lifestyles to their patients in terms of nutrition, physical activity and sleep hygiene. Learning to what extent a longitudinal integration and interprofessional education of Culinary Medicine will impact the dietary habits of populations and curb the alarming incidence rates of obesity will take time. However, we cannot wait longer to implement such medical practice since we know that poor nutrition is the major trigger of this epidemic of obesity.

With recognition that nutrition plays an integral role in human development and in the prevention and treatment of disease, recent initiatives in the United States are important to mention. A clear example of the importance of the problem was the development of the “Food is Medicine” project launched at the September 2022 “White House Conference on Hunger, Nutrition and Health” as a call to action to “end hunger and reduce the prevalence of chronic disease in the US by 2030” (5). Similarly, the National Institutes of Health has launched a large study called “Nutrition for Precision Health powered by All of Us” to improve our understanding of how individual human biology and molecular pathways influence relationships among diet and environmental, social, and behavioral factors to influence health (6) Such efforts will continue to impact our understanding of the relationship between nutrition and health. The resulting knowledge will need to be taught to physicians and health care professionals to translate scientific discoveries to populations and truly use food as medicine.

Since the epidemic of obesity has been triggered by a change in the environment, better training of our health care providers including Culinary Medicine will be an initial step to reverse the problem. In parallel, public health systems need to reverse some of the “obesogenic” nutritional practices by imposing novel public health messages and policies. This will only be possible by working closely with nutrition companies who have developed some of these unhealthy foods and can of course also produce healthier foods if incentivized. To protect our children as much as possible from our obesogenic built environment, it is essential to create a home environment that promotes healthy habits. Encourage a balanced diet by providing nutritious meals and limiting the availability of unhealthy snacks. Additionally, prioritize regular physical activity by integrating fun and engaging exercises into the daily routine and reducing sedentary activities like screen time. Educating children about the importance of healthy eating and active living can also empower them to make better choices outside the home. But of course, these healthier behaviors must come from solid scientific evidence that they make a difference and dispersed by well-educated healthcare professionals. It will also be important to develop simple biomarkers of optimal nutrition practices. The implementation of Clinical Medicine represents a first step in the right direction as summarized in the paper by Vasques and cols. (4).
